# Numerical Simulation on Seismic Response of the Filled Joint under High Amplitude Stress Waves Using Finite-Discrete Element Method (FDEM)

**DOI:** 10.3390/ma10010013

**Published:** 2016-12-27

**Authors:** Xiaolin Huang, Qi Zhao, Shengwen Qi, Kaiwen Xia, Giovanni Grasselli, Xuguang Chen

**Affiliations:** 1 Key Laboratory of Shale Gas and Geoengineering, Institute of Geology and Geophysics, Chinese Academy of Sciences, Beijing 100029, China; huangxiaolin@mail.iggcas.ac.cn; 2 Department of Civil Engineering, University of Toronto, Toronto, ON M5S 1A4, Canada; q.zhao@mail.utoronto.ca (Q.Z.); kaiwen.xia@utoronto.ca (K.X.); giovanni.grasselli@utoronto.ca (G.G.); 3 College of Engineering, Ocean University of China, Qingdao 266100, China; chenxuguang1984@ouc.edu.cn

**Keywords:** high amplitude stress wave, filled joint, amplitude attenuation, particle crushing, grain size reduction, FDEM

## Abstract

This paper numerically investigates the seismic response of the filled joint under high amplitude stress waves using the combined finite-discrete element method (FDEM). A thin layer of independent polygonal particles are used to simulate the joint fillings. Each particle is meshed using the Delaunay triangulation scheme and can be crushed when the load exceeds its strength. The propagation of the 1D longitude wave through a single filled joint is studied, considering the influences of the joint thickness and the characteristics of the incident wave, such as the amplitude and frequency. The results show that the filled particles under high amplitude stress waves mainly experience three deformation stages: (i) initial compaction stage; (ii) crushing stage; and (iii) crushing and compaction stage. In the initial compaction stage and crushing and compaction stage, compaction dominates the mechanical behavior of the joint, and the particle area distribution curve varies little. In these stages, the transmission coefficient increases with the increase of the amplitude, i.e., peak particle velocity (PPV), of the incident wave. On the other hand, in the crushing stage, particle crushing plays the dominant role. The particle size distribution curve changes abruptly with the PPV due to the fragments created by the crushing process. This process consumes part of wave energy and reduces the stiffness of the filled joint. The transmission coefficient decreases with increasing PPV in this stage because of the increased amount of energy consumed by crushing. Moreover, with the increase of the frequency of the incident wave, the transmission coefficient decreases and fewer particles can be crushed. Under the same incident wave, the transmission coefficient decreases when the filled thickness increases and the filled particles become more difficult to be crushed.

## 1. Introduction

Joints commonly exist in the rock mass, which have an important effect on the mechanical behavior of the rock mass [1,2]. Generally, a joint can slow down and attenuate the stress wave [3]. The study of the seismic response of the joint is one of the main tasks in rock dynamics, and numerical methods are often commercial and feasible in the study of this subject.

Numerous field investigations showed that two types of naturally occurring joints commonly exist, i.e., the unfilled joint and the filled joint. The unfilled joint, with no material filled in the joint gap, is often treated as an interface with zero thickness in the numerical models. Considering different joint constitutive models, many researchers have investigated the propagation of the stress wave through unfilled joints, using discontinuous deformation analysis (DDA) [4], numerical manifold method (NMM) [5], particle manifold method (PMM) [6], distinct lattice spring model (DLSM) [7], and the discrete element method (DEM) [8,9,10,11,12,13,14,15].

**Figure 1:**
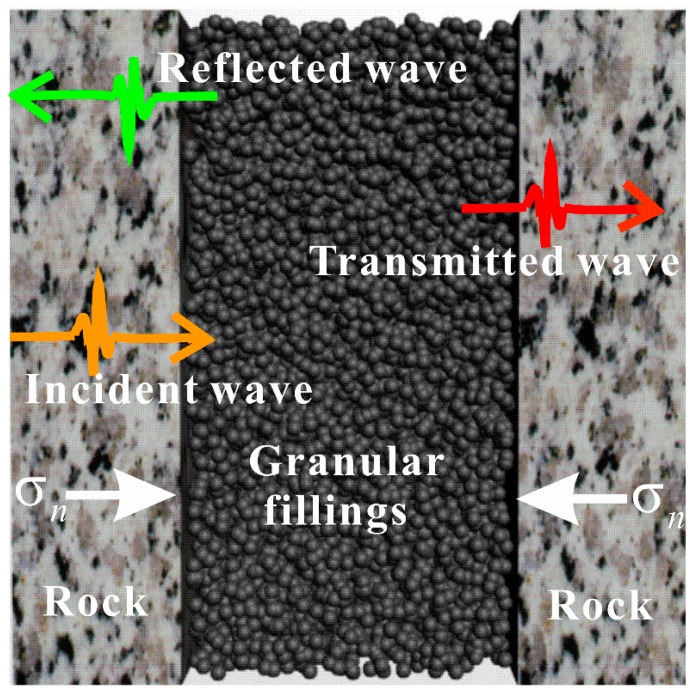
Schematic view of a filled joint loaded by stress waves (adapted from [23]).

The filled joint, on the other hand, is often filled with granular materials such as sand, clay, and weathered rock as shown in Figure 1. The fillings may have a thickness up to several centimeters, which have a noticeable influence on the mechanical behavior of the joint [16,17]. It has been also found that the seismic response of the filled joint depends on the density, thickness, and mechanical behavior of the fillings [18,19]. Hence, the filled joint cannot be simply treated as a zero-thickness interface in the numerical models. Compared with the case of the unfilled joint, there were very limited studies regarding the numerical simulation of the seismic response of filled joints reported in the literature. In [6], the researchers conducted the numerical simulation on the propagation of the stress wave through the filled joints using PMM. A thin layer of bonded particles were welded on the background rock to model the filled joint. The effect of the filled mass and joint thickness on the wave attenuation was discussed. In their study, the bonded particle medium was actually a thin layer of elastic medium which did not undergo plastic deformation regardless of the amplitude of the stress waves. Nevertheless, this elastic deformation assumption is far from reality when the geological properties of the filled joints are taken into account. As shown in Figure 1, the granular fillings suffer the dynamic compressive stress *σ*_n_ resulting from the combined influence of the incident, reflected, and transmitted waves. Experimental data tested by the rock split Hopkinson pressure bar (SHPB) showed that the filled joints endured nonlinear deformation due to the plastic flow and compaction of the fillings [20,21]. Therefore, the elastic-plastic model is likely to be more suitable to describe the deformation behavior of the filled joint. Based on DEM, [22] used a thin layer of particles without bond strength to simulate the filled joint and accurately reproduced the SHPB test on the propagation of the low amplitude stress wave through a filled artificial joint.

On the other hand, field test data has shown that the practical blasting stress waves often have high amplitudes [24,25]. Near the blasting source, the high amplitude of the stress wave often causes the filled granular material to bear a high stress state. It has been found that the granular particles, such as quartz sand, can be crushed under the high compressive stress in the laboratory tests [26]. Recently, some interesting experimental phenomena were reported on regarding the seismic response of the filled joint under high amplitude stress waves, by making full use of the high-strength metallic SHPB. In [23], researchers observed the dynamic weakening and acoustic fluidization of the granular fault gouge under high amplitude stress waves. The researchers in [27] conducted laboratory tests on the response of artificial filled joints under high amplitude stress waves. It was found that the particle crushing has a very important influence on the transmission behavior of stress waves through filled joints. However, up to now, few numerical models were reported to study the effect of the particle crushing on the propagation of high amplitude stress waves through filled joints, and the main purpose of this paper is to numerically investigate this subject.

This paper is structured as follows. Section 1 reviews the previous numerical studies on the propagation of stress waves through rock joints and analyzes the limitations in these studies. Section 2 introduces the FDEM modelling on the seismic response of the filled joint under high amplitude stress waves. Section 3 shows the numerical results in detail and these results are discussed in Section 4. Conclusions are given in Section 5.

## 2. FDEM Modelling

### 2.1. Principles of FDEM

FDEM is an advanced numerical method which combines continuum mechanics with DEM algorithms to simulate multiple interacting deformable solids [28]. In FDEM, the elastic deformation of discrete bodies is employed in a traditional finite element method (FEM), while the nucleation of new fractures and the interaction between discrete bodies are captured by DEM. A solid is initially discretized using a triangular FEM mesh, and cohesive joint elements are inserted between adjacent FEM elements. Before the failure of the simulated material, the model experiences linear elastic deformation. When an excessive amount of deformation occurs, the cohesive joint elements can be deformed through open or slip and eventually break. As a consequence, the original meshed solids can be cracked into new independent ones. After that, the numerical simulation employs the DEM algorithm to capture the interaction between solids. There are two types of material properties involved in FDEM models, i.e., macro- and micro-properties. Macro-properties including Young’s modulus (*E*) and Poisson’s ratio (*υ*) are used to describe the elastic deformation of the discrete bodies; whereas micro-properties describing the strength of the cohesive joint elements control the cracking behavior of the material, i.e., tensile strength *f_t_*, cohesion strength *c*, model I fracture energy *G_I_*, and model II fracture energy *G_II_* [29,30,31,32,33]. The cohesive joint model in FDEM is actually derived from the Mohr-Coulomb type behavior of the material with maximum tensile strength cut-off, except that two fracture energy properties (*G_I_* and *G_II_*) are introduced. After the breakage of the cohesive joint elements, the newly created surfaces can interact with each other according to the contact algorithm, through the normal and tangential contact forces which are described by the normal, tangential penalties (*p_n_*, *p_t_*) and the friction coefficient of the fracture surface *μ_f_*. More detailed definitions of the above-mentioned parameters can be found in [29,30,31,32,33].

### 2.2. Model Description

**Figure 2:**
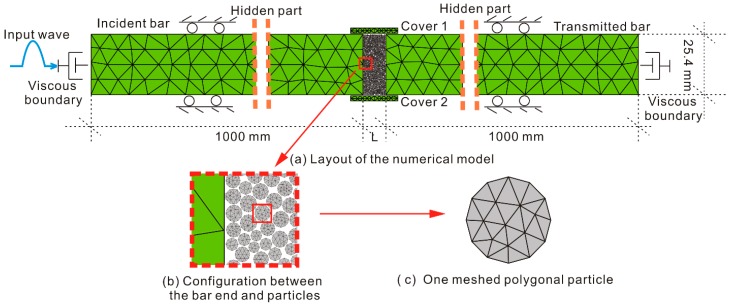
Layout of the FDEM model.

In this paper, we focus on 1D longitude stress wave problems. Owing to the computational capacity, FDEM models only have limited length in this paper. Figure 2 shows the FDEM model which consists of an incident bar, a transmitted bar, two covers, and a thin layer of polygonal particles. The *x-o-y* coordinate system was introduced into the model, with the origin located at the geometric center of the model. Both the incident and transmitted bars have a width of 25.4 mm and a length of 1000.0 mm. The two bar ends that contact with the particles are planar (Figure 2b). Similar to the configuration in [22], a thin layer of particles without bond strength were initially sandwiched between the incident and transmitted bars to simulate the joint fillings. The centroids of polygonal particles are distributed randomly in the joint gap. The particle size uniformly ranges from 0.6 to 0.96 mm, with a mean size of 0.78 mm. The porosity of the fillings is 27.1%, which is close to the porosity of the natural dense sand. The maximum mesh size was set as 0.3 mm. Two covers, each with a length of 20.0 mm and a width of 2.0 mm, were placed on the top and bottom of the filled layer and were meshed with a maximum element size of 1.0 mm. Previous studies showed that the ratio of the maximum mesh size to the wavelength, *γ*, should be less than 1/8–1/12 in order to guarantee the simulation accuracy of the wave propagation problem [8]. The P-wave velocity of the rock bars was calculated as 4758.3 m/s (based on the assigned properties in the following section). In this paper, the maximum mesh size of the bars was taken as 8.0 mm. Therefore, *γ* can be calculated as around 1/50 according to the minimum incident wavelength of 396.0 mm (based on the largest frequency of 12.0 kHz involved in the following section).

### 2.3. Boundary Conditions

Note that the FDEM model in Figure 2 only has limited length. When arriving at the truncated boundaries, stress waves will be reflected back from the bar ends, which then interfere with the recording of the wave signals. To eliminate this defect, the left end of the incident bar and the right end of the transmitted bar were both set as the viscous boundary (no reflection boundary). Because only the longitude waves are considered in this paper, both upper and lower boundaries of the bars were fixed in the y-direction but free in the x-direction. The two covers were fixed in both x- and y-directions to prevent the outflow of the particles when loaded by the stress waves. Positive parts of the sinusoidal waves *v* = *A*sin2*πft* (0 ≤ *t* ≤ 1/(2*f*)) were adopted as the incident wave, where *v* denotes the particle velocity; *A* is the particle peak velocity (PPV); *f* is the frequency; and *t* is the time variable. The incident waves were inputted at the left end of the incident bar.

### 2.4. Assignment of Model Properties

**Figure 3:**
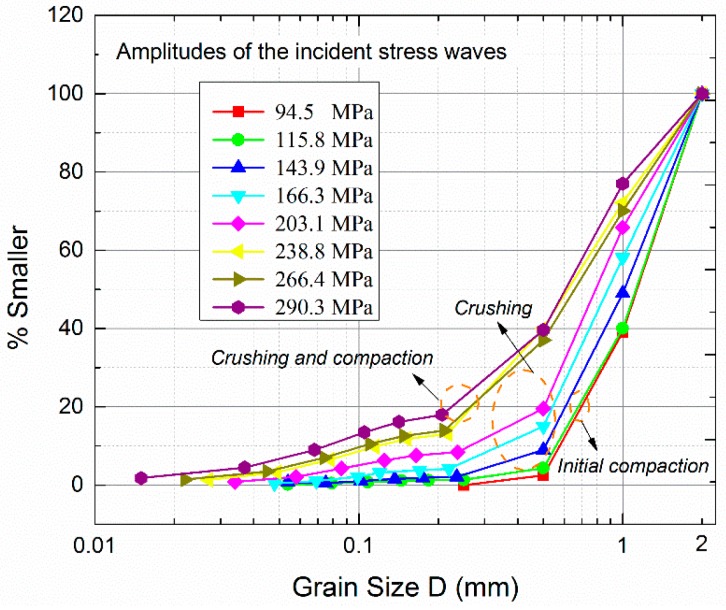
The grain size distributions of the filled quartz sand after loading by stress waves with different amplitudes (adapted from [27]).

During simulation, bars and covers were treated as pure elastic bodies which cannot produce failure when loaded, while the filled particles can be crushed. The property of the covers was set as that of the bars. Usually, macro-properties such as Young’s modulus *E* and Poisson’s ratio *υ* can be directly acquired from laboratory tests. Table 1 lists the macro-properties in the calculation. Nevertheless, there exists limited experience on estimating dynamic micro-properties in FDEM. In this study, the assignment of FDEM micro properties of the filled particles referred to that of the granite according to [30]. The model II fracture energy was taken as 2500.0 J/m^2^, with two times of model I fracture energy. The micro tensile strength and cohesion strength were taken as 12.7 MPa and 50.0 MPa, respectively. From SHPB tests on the artifical filled joint, [27] found that the filled sand particles experienced three different deformation stages depending on the amplitude of the incident wave, i.e., initial compaction stage, crushing stage, and crushing and compaction stage. Figure 3 shows the influence of the three different stages on the grain size distribution of the filled sand. When the wave amplitude was relatively small (≤115.8 MPa), the grain size curve basically stayed invariant regardless of the increment of the wave amplitude, which indicates that the particles were only compacted. As the wave ampiltude ranged from 115.8 to 238.8 MPa, the grain size curve varied obviously and the number of smaller particles significantly increased. It can be concluded that numerous partilces were crushed into smaller ones in this stage. When the wave amplitude was larger than 238.8 MPa, the grain size curve only changed slightly with the increase of the wave amplitude, which reveals that only a few particles were crushed. Nevertheless, the numerical results considering the micro tensile strength of 12.7 MPa and micro cohesion strength of 50.0 MPa showed that most of the particles were crushed only when the incident wave had a relatively small amplitude (2.0 m/s). That is, the grain size curve changed obviously once the amplitude of the incident wave was very small. It is thus difficult to reproduce the complete three deformation stages of the filled particles observed by [27], when the micro tensile strength and cohesive strength were assigned according to [30].

To obtain better results, a feasible method is to choose the micro properties by trial and error until the three different deformation stages observed in the laboratory tests can be basically reproduced by the FDEM model. After many trials, it was found that the complete three deformation stages of the filled particles can be reproduced when the dynamic micro tensile strength was taken as 114.0 MPa and the dynamic cohesion was set as 228.0 MPa. Meanwhile, the normal penalty was taken as ten times the Young’s modulus of the particle and the shear penalty was equal to the Young’s modulus [28]. All involved micro properties are listed in Table 2.

## 3. Results

In this paper, we are only concerned about the transmission characteristic of the stress wave. Two monitoring points near (−900.0 mm, 0.0 mm) and (100.0 mm, 0.0 mm) were set to record the incident and transmitted waves, respectively. For comparison purposes, two types of models were adopted in the simulation: the crushing model and the non-crushing model. For the non-crushing model, the strength properties of the filled particles were set as very large values in order to stop the particles from crushing.

### 3.1. Transmitted Waveforms

**Figure 4:**
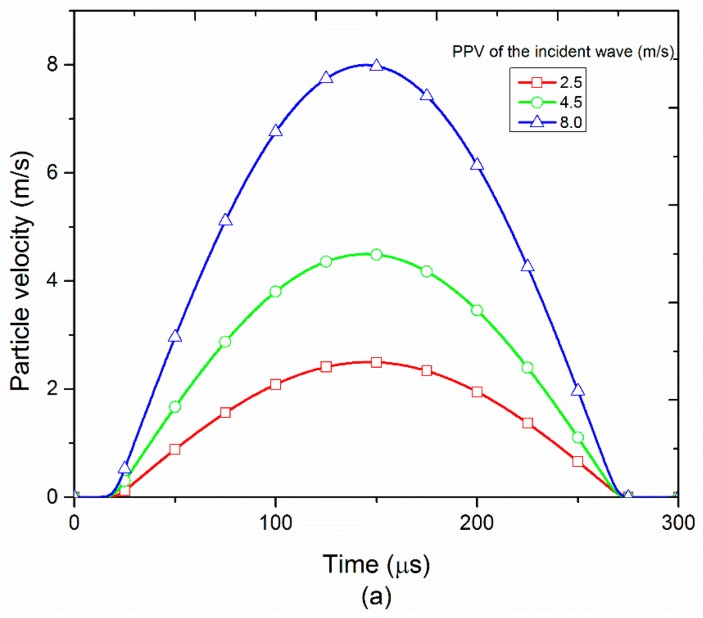

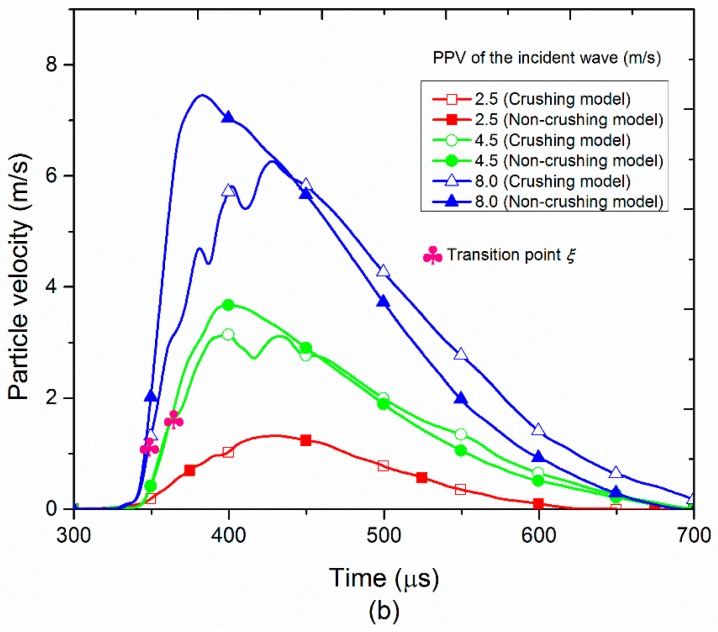
(**a**) Incident waves with different PPVs; (**b**) Corresponding transmitted waves obtained by the crushing model and the non-crushing model, respectively.

Figure 4a shows the monitored incident waves with PPV of 2.5 m/s, 4.5 m/s, and 8.0 m/s. The incident waves have the same *f* of 2.0 kHz and the filled joint has a thickness of 10.0 mm. Figure 4b shows the corresponding transmitted waveforms which were plotted together with the same arrival time for convenient comparison. It can be seen that the transmitted waves obtained by the crushing and non-crushing models are the same when the PPV is 2.5 m/s. When the PPV reaches 4.5 m/s and 8.0 m/s, the transmitted wave resulting from the crushing model initially coincides with that by the non-crushing model; however, it deviates after passing through a transition point *ξ*. Meanwhile, the slope of the transmitted waveform of the crushing model abruptly decreases after *ξ*, and fluctuations appear in the transmitted waveform. The transmitted wave of the crushing model has a smaller amplitude than that of the non-crushing model.

### 3.2. The Crush of Filled Particles and Its Effect on the Transmission Coefficient

#### 3.2.1. Influence of the PPV of the Incident Wave

**Figure 5:**
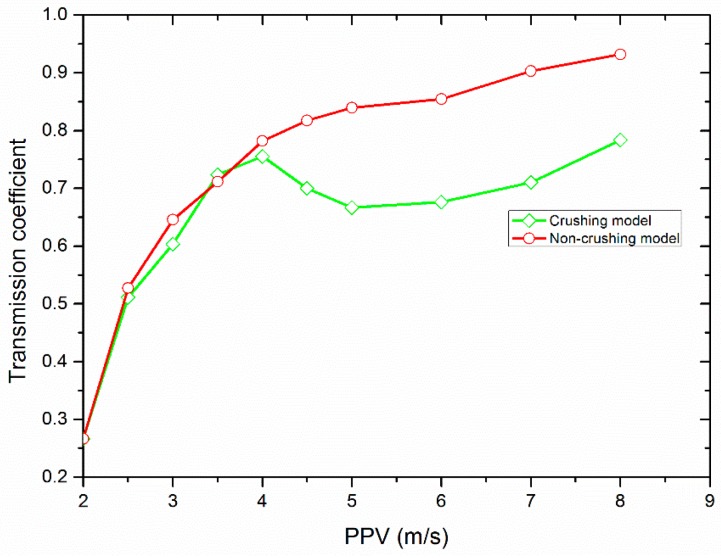
Variation of the transmission coefficient with the PPV of the incident wave.

The transmission coefficient defined as the ratio of the peak value of the transmitted wave to that of the incident wave is a significant index to evaluate the amplitude attenuation of the stress wave though the filled joint. Figure 5 shows the variation of the transmission coefficient versus the PPV when *f* is 2.0 kHz. It can be observed that the transmission coefficient obtained by the non-crushing model nonlinearly increases with the PPV of the incident wave. However, the transmission coefficient resulting from the crushing model first increases with the PPV, and after achieving a peak value it decreases, and finally it increases again from a valley. The transmission coefficient as a function of PPV follows the same pattern for the non-crushing model and the crushing model when the PPV of the incident wave is less than about 2.5 m/s.

**Figure 6:**
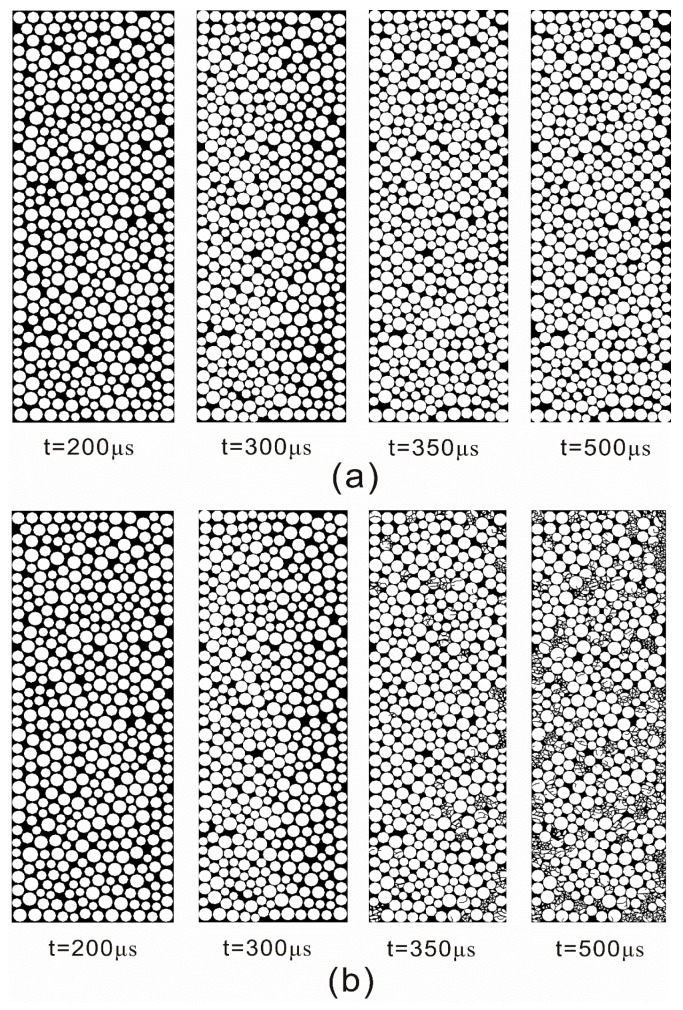
Snapshots of the configuration change of the filled particles at different times. (**a**) The result of the non-crushing model; (**b**) The result of the crushing model.

Figure 6 show the configuration change of the filled particles at different times when the incident wave has a PPV of 8.0 m/s. It can be found that for the non-crushing model, the filled particles were only compacted when loaded by stress waves (Figure 6a). However, for the crushing model, the filled particles were first compacted and then crushed (Figure 6b).

**Figure 7:**
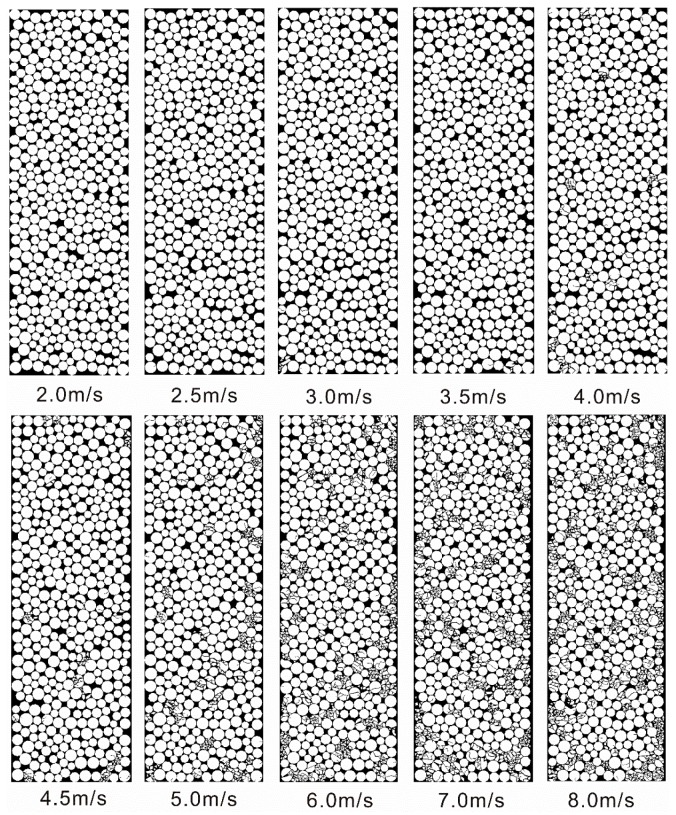
The configuration change of the filled particles after loading by the stress waves with different PPVs.

Figure 7 show the configuration change of the filled particles for the crushing model, after being loaded by the stress waves with different PPVs. It can be seen that the particles were not crushed when the PPV is less than 2.5 m/s. When the PPV is 3.0 m/s, some crushed particles appear in the left bottom image of the filled particles. When the PPV ranges from 3.5 m/s to 6.0 m/s, the number of crushed particles increases abruptly, while this variation trend becomes gentle when the PPV is larger than 6.0 m/s.

**Figure 8:**
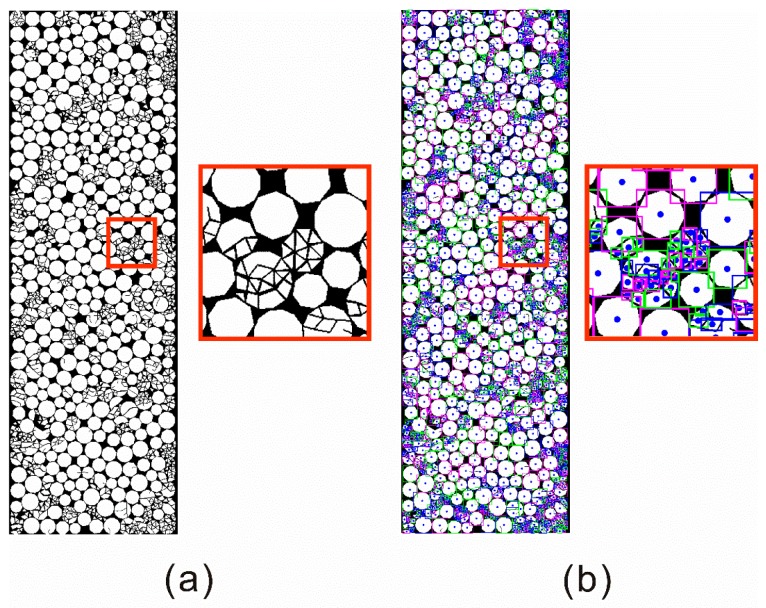
Details of the method to obtain the particle area distribution. (**a**) The original image of the filled particles; (**b**) The processed picture after each polygonal particle was marked by a rectangle with a dot in the center.

To quantitatively investigate the configuration change of the filled particles, a custom Matlab code was used to conduct the grain area distribution analysis, and the method is illustrated in Figure 8. The image of the filled joint layer (visualized using an open source software, Paraview, Sandia National Laboratories, Albuquerque, NM, USA) was first converted to a black-white image (see Figure 8a), in which white areas denote particles and the black areas represent void space. Then each isolated white area was identified as an independent polygonal particle whose area can be calculated. According to *A*_r_ = *l^2^A*_p_, the real area of the particle *A*_r_ can be obtained, where *A*_p_ is the pixel area and *l* is the length of each pixel. Finally, we carry out the statistical analysis on the particle size distribution.

**Figure 9:**
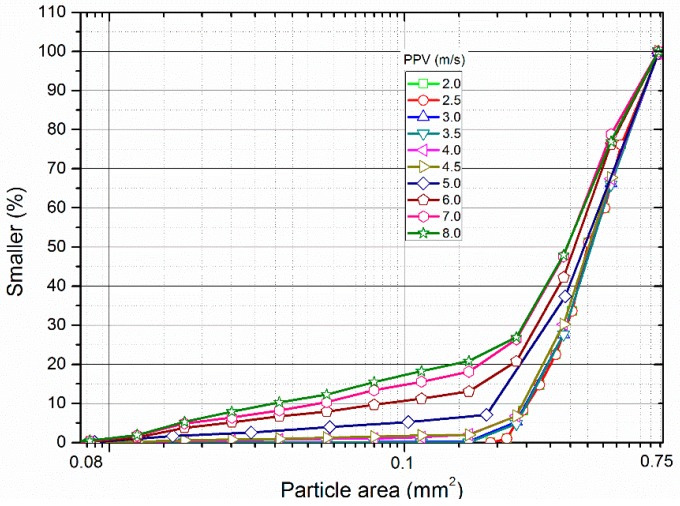
The particle area distribution of the filled particles after loading by stress waves with different PPVs.

Figure 9 shows the particle area distribution of the filled particles after being loaded by the stress wave with different PPVs. It can be seen that the curves are steep and coincide with each other when the PPV is small (<4.5 m/s). There exist few particles with area less than 0.3 mm^2^. With the increase of the PPV, the number of particles with areas less than 0.3 mm^2^ increases. When the PPV is larger than 4.5 m/s, the curve has evident changes and smaller particles become more numerous. As the PPV ranges from 7.0 to 8.0 m/s, the change of the curves is not obvious.

#### 3.2.2. Influence of the Frequency of the Incident Wave

**Figure 10:**
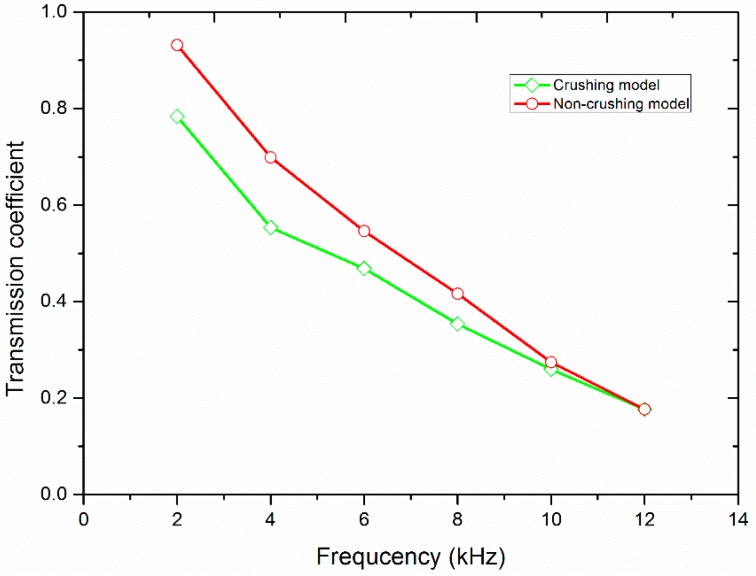
The variation of the transmission coefficient with the frequency of the incident wave.

Figure 10 shows the variation of the transmission coefficient with the frequency of the incident wave. The filled joint has a thickness of 10.0 mm and the PPV of the incident wave is 8.0 m/s. The crushing model and the non-crushing model were both examined. It can be found that the transmission coefficient curves obtained by the two models both decrease with an increase of the frequency. Nevertheless, there exist some differences between the two curves. The transmission coefficient resulting from the crushing model is smaller than that of the non-crushing model. With the increase of the frequency, the magnitude difference between the two results becomes smaller and smaller. When the frequency is 10.0 kHz, the transmission coefficients calculated from the two models have the same magnitude.

**Figure 11:**
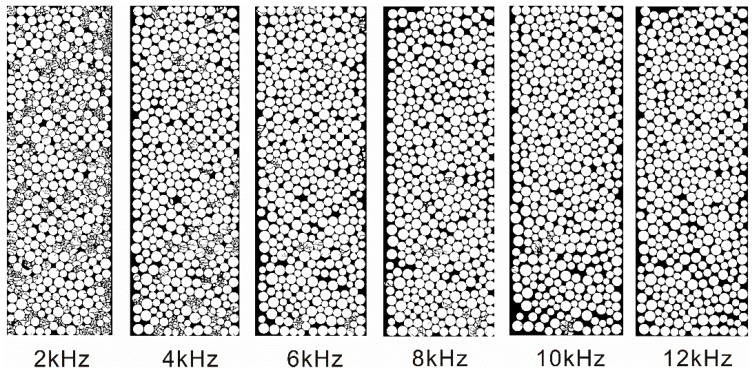
The configuration change of the filled particles after loading by the stress waves with different frequencies.

Figure 11 show the configuration change of the filled particles. It can be seen that the amount of crushed particles decreases abruptly with the increment of the frequency. No crushed particles exist when the frequency is 12.0 kHz.

**Figure 12:**
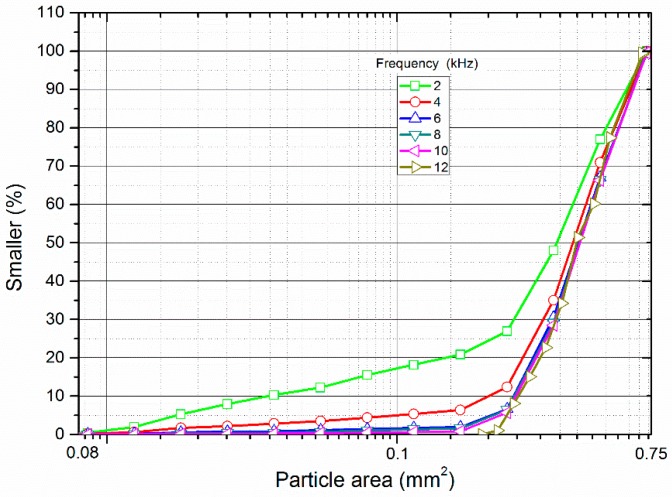
The particle area distribution of the filled particles after loading by stress waves with different frequencies.

Figure 12 shows particle area distribution of the filled particles after loading by the stress wave with different frequencies. It can be observed that the curve becomes steeper with the increment of the frequency. When the frequency ranges from 2.0 to 6.0 kHz, the curve changes obviously. However, it only has a slight change when the frequency is larger than 6.0 kHz. Such a result indicates that the stress wave with low frequency can induce more crushed particles than that with high frequency.

#### 3.2.3. Influence of the Filled Thickness

**Figure 13:**
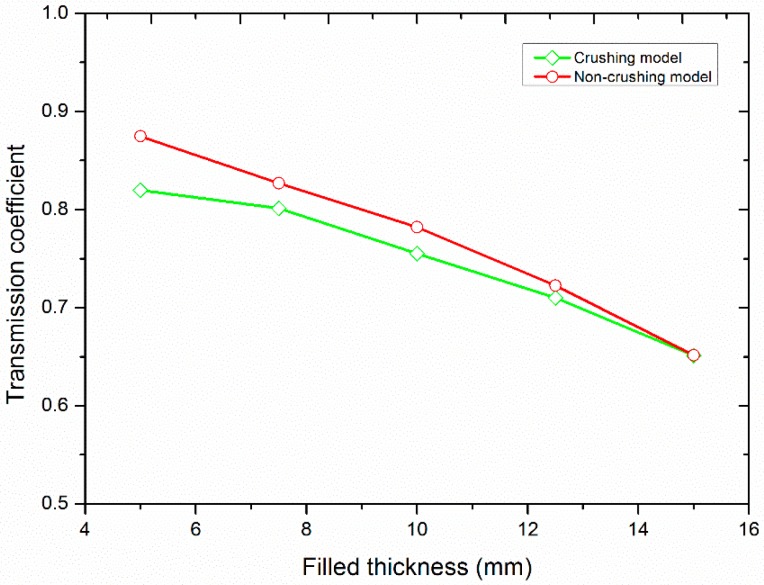
The variation of the transmission coefficient with the filled thickness.

In this section, the incident waveform was fixed while the thickness of the filled joint varied. The incident wave had a PPV of 4.0 m/s and a frequency of 2.0 kHz. Figure 13 shows the variation of the transmission coefficient versus the thickness of the filled joint, taking into account the crushing model and the non-crushing model. It can be seen that the transmission coefficient obtained by the two models both decrease with an increase of the filled thickness. Generally, the transmission coefficient obtained by the crushing model is smaller than that obtained by the non-crushing model. With an increase of the thickness, the magnitude difference between them becomes smaller. When the thickness is 15.0 mm, the transmission coefficient obtained by the two models is the same.

**Figure 14:**
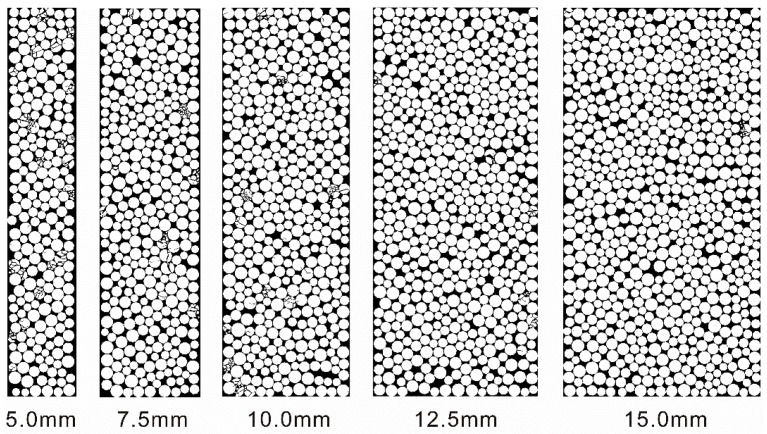
The configuration change of the filled joint with different thicknesses after loading by the stress waves.

Figure 14 shows the configuration change of the filled joint with different thicknesses after loading by the stress wave. It can be found that more crushed particles exist in the thin filled joint than that in the thick filled joint. With the increase of the filled thickness, the filled particles become more difficult to crush. When the thickness reaches 15.0 mm, there are few crushed particles. Because the thickness (or number) of the filled particles varies, we did not analyze the particle area distribution in this section.

## 4. Discussion

When impinging on a filled joint, only one part of the stress wave can pass through, and according to the SHPB testing theory, the transmitted stress wave is actually the dynamic compressive stress *σ*_n_ that induces the deformation of the filled particles. Due to *σ*_n_, plastic flow and compaction were produced in the filled particles (see Figure 6). This process causes the density and the stiffness of the filled material to increase. It has been found that the increase of both the density of the fillings and stiffness of the filled joint can allow more waves to propagate through the filled joint [19]. For the non-crushing model, dynamic compaction is only experienced in the filled particles (see Figure 6a). The larger the PPV of the stress wave, the much denser the compacted filled particles. As a result, the transmission coefficient always increases with the PPV (see Figure 5). However the grannular material in a natural filled joint such as sand has limited strength. When the PPV is small (<4.0 m/s), the induced transmitted waves can only produce a relative small amount of crushed particles (see Figure 7). Hence, the particle area distribution curve changes little (see Figure 9). We call this process the initial compaction stage. In this stage, the transmission coefficient calculated by the crushing model has the same variation trend as that by the non-crushing model (see Figure 5). However much more particles in the crushing model were crushed when the PPV is large (>4.0 m/s) (see Figure 6b). Due to the crushing event, more particles with smaller area were produced, which results in the abrupt increase of the area percentage of small particles (see Figure 9). Also, fluctuations exist in the transmitted waveforms, due to the crushing behavior (see Figure 4b). The crushing of the particles has two effects on the propagation of the stress wave: (i) it causes the stiffness of the filled joint to decrease; and (ii) it consumes part of the wave energy. In general, the incident wave with large PPV can produce more crushed particles (see Figure 7). Therefore, the transmission coefficient decreases with the increment of the PPV (see Figure 5). We named this process as the crushing stage. When the PPV is very large (>6 m/s), the particle area curve changes little (see Figure 9). It indicates that the filled particles were mainly compacted accompanied by crushing. Therefore, the transmission coefficient increases with the PPV again (see Figure 5). We call this process the crushing and compaction stage. It can be found that the results of the crushing model in Figure 5 and Figure 9 are very similar with those observed by [27] in the laboratory, which indicates that the FDEM model is capable of simulating the seismic response of the filled joint under high amplitude stress waves.

It has been found that the filled joint can allow more incident waves with low frequency pass though than those with high frequency [22]. That is, the transmission coefficient decreases with the increase of the frequency of the incident wave. When the frequency of the incident wave is relatively small, the amplitude of the induced transmitted wave is large enough to lead to the crushing of the filled particles. Therefore, the transmission coefficient of the crushing model is smaller than that of the non-crushing model under the same frequency (see Figure 10). However, the amplitude of the transmitted stress wave decreases with the increase of the frequency, which causes the abrupt decrease of the number of the crushed particles (see Figure 11). Consequently, the particle area distribution curve moves downward with the increase of the frequency (see Figure 12). The transmission coefficient of the crushing model is gradually closer to that of the non-crushing model with the increase of the incident wave frequency (see Figure 10). When the frequency is 12 kHz, there are no particles crushed. Thus, the transmission coefficient of the two models is the same.

It has been also found that the transmission coefficient decreases with the increment of the filled thickness [22]. That is, the transmitted wave through the thin joint has a higher amplitude than that through the thick joint under the same incident wave. When the filled thickness is small, the amplitude of the transmitted wave is high enough to cause the crushing of the filled particles. Therefore, the transmission coefficient of the crushing model is smaller than that of the non-crushing model (see Figure 13). With the increase of the filled thickness, the number of the crushed particles decreases (see Figure 14). And the difference of the transmission coefficient by the two models gradually becomes smaller and finally is the same.

## 5. Conclusions

The research on the propagation of the stress wave through the joined rock mass is of great significance in blasting engineering and geophysical exploration. Using FDEM, we simulated the seismic response of the filled joint under high amplitude stress waves and addressed some important conclusions. Under the stress wave, the filled particles mainly experience three deformation stages depending on the amplitude of the incident wave, i.e., initial compaction stage, crushing stage, and crushing and compaction stage. In the initial compaction stage and crushing and compaction stage, the filled particles are mainly compacted and the particle area distribution curve changes slightly. The transmission coefficient increases with the increase of the PPV. However, the particle crushing plays the dominant role in the crushing stage and the particle size distribution curve changes abruptly in this stage due to the significantly increased amount of small fragments. The crushing stage consumes part of the wave energy and reduces the stiffness of the filled layer. As a result, the transmission coefficient decreases with the increase of the PPV in this stage. When the frequency of the incident wave increases, the transmission coefficient decreases and fewer particles are crushed. Under the same incident wave, the transmission coefficient decreases and the filled particles become more difficult to be crushed with the increment of the filled thickness.

## Figures and Tables

**Table 1:** Macro properties of the FDEM model.

Parameters	Rock Bars (Granite)	Particles (Fused Quartz Sand)
Young’s modulus *E* (GPa)	60.0 ^a^	72.0 ^c^
Poisson’s ratio *υ*	0.20 ^b^	0.17 ^c^
Density *ρ* (kg/m^3^)	2650.0 ^a^	2200.0 ^c^
Friction coefficient of the intact material *μ_i_*	0.25 ^b^	0.25 ^b^

^a^ From [20]; ^b^ Estimated by experience; ^c^ From http://www.quartz.com/gedata.html.

**Table 2:** Micro properties of the FDEM model.

Parameter	Value
Tensile strength *f_t_* (MPa)	114
Cohesion strength *c* (MPa)	228
Mode I fracture energy *G_I_* (J/m^2^)	1250
Mode II fracture energy *G_II_* (J/m^2^)	2500
Friction coefficient of the fracture *μ_f_*	0.5
Normal contact penalty, *p_n_* (GPa/m)	720
Tangential contact penalty, *p_t_* (GPa/m)	72

## References

[1] Goodman R.E. (1976). Methods of Geological Engineering in Discontinuous Rocks.

[2] Sun G.Z. (1988). Rock Mass Structure Mechanics.

[3] King M., Myer L., Rezowalli J. (1986). Experimental studies of elastic-wave propagation in a columnar-jointed rock mass. Geophys. Prospect..

[4] Gu J., Zhao Z. (2009). Considerations of the discontinuous deformation analysis on wave propagation problems. Int. J. Numer. Anal. Methods Geomech..

[5] Zhao G.F., Zhao X.B., Zhu J.B. (2014). Application of the numerical manifold method for stress wave propagation across rock masses. Int. J. Numer. Anal. Methods Geomech..

[6] Zhao J., Sun L., Zhu J.B. (2012). Modelling p-wave transmission across rock fractures by particle manifold method (pmm). Geomech. Geoeng..

[7] Zhu J.B., Zhao G.F., Zhao X.B., Zhao J. (2011). Validation study of the distinct lattice spring model (DLSM) on p-wave propagation across multiple parallel joints. Comput. Geotech..

[8] Cai J., Zhao J. (2000). Effects of multiple parallel fractures on apparent attenuation of stress waves in rock masses. Int. J. Rock Mech. Min. Sci..

[9] Chen S., Zhao J. (1998). A study of udec modelling for blast wave propagation in jointed rock masses. Int. J. Rock Mech. Min. Sci..

[10] De Lemos J.A.S.V. (1997). A Distinct Element Model for Dynamic Analysis of Jointed Rock with Application to Dam Foundations and Fault Motion.

[11] Resende R., Lamas L.N., Lemos J.V., Calçada R. (2010). Micromechanical modelling of stress waves in rock and rock fractures. Rock Mech. Rock Eng..

[12] Deng X.F., Zhu J.B., Chen S.G., Zhao J. (2012). Some fundamental issues and verification of 3DEC in modeling wave propagation in jointed rock masses. Rock Mech. Rock Eng..

[13] Zhao J., Cai J.G., Zhao X.B., Li H.B. (2007). Dynamic model of fracture normal behaviour and application to prediction of stress wave attenuation across fractures. Rock Mech. Rock Eng..

[14] Zhao X.B., Zhao J., Cai J.G., Hefny A.M. (2008). Udec modelling on wave propagation across fractured rock masses. Comput. Geotech..

[15] Zhu J.B., Deng X.F., Zhao X.B., Zhao J. (2012). A numerical study on wave transmission across multiple intersecting joint sets in rock masses with udec. Rock Mech. Rock Eng..

[16] Barton N. (1974). A Review of the Shear Strength of Filled Discontinuities in Rock.

[17] Sinha U., Singh B. (2000). Testing of rock joints filled with gouge using a triaxial apparatus. Int. J. Rock Mech. Min. Sci..

[18] Wu W., Li J.C., Zhao J. (2013). Seismic response of adjacent filled parallel rock fractures with dissimilar properties. J. Appl. Geophys..

[19] Zhu J.B., Perino A., Zhao G.F., Barla G., Li J.C., Ma G.W., Zhao J. (2011). Seismic response of a single and a set of filled joints of viscoelastic deformational behaviour. Geophys. J. Int..

[20] Li J.C., Ma G.W. (2009). Experimental study of stress wave propagation across a filled rock joint. Int. J. Rock Mech. Min. Sci..

[21] Wu W., Li J.C., Zhao J. (2012). Loading rate dependency of dynamic responses of rock joints at low loading rate. Rock Mech. Rock Eng..

[22] Huang X.L., Qi S.W., Williams A., Zou Y., Zheng B.W. (2015). Numerical simulation of stress wave propagating through filled joints by particle model. Int. J. Solids Struct..

[23] Xia K.W., Huang S., Marone C. (2013). Laboratory observation of acoustic fluidization in granular fault gouge and implications for dynamic weakening of earthquake faults. Geochem. Geophys. Geosyst..

[24] Khandelwal M., Singh T. (2007). Evaluation of blast-induced ground vibration predictors. Soil Dynam. Earthq. Eng..

[25] Singh P.K. (2002). Blast vibration damage to underground coal mines from adjacent open-pit blasting. Int. J. Rock Mech. Min. Sci..

[26] Omidvar M., Iskander M., Bless S. (2012). Stress-strain behavior of sand at high strain rates. Int. J. Impact Eng..

[27] Huang X., Qi S., Xia K., Zheng H., Zheng B. (2016). Propagation of high amplitude stress waves through a filled artificial joint: An experimental study. J. Appl. Geophys..

[28] Munjiza A., Owen D., Bicanic N. (1995). A combined finite-discrete element method in transient dynamics of fracturing solids. Eng. Comput..

[29] Lisjak A., Grasselli G., Vietor T. (2014). Continuum–discontinuum analysis of failure mechanisms around unsupported circular excavations in anisotropic clay shales. Int. J. Rock Mech. Min. Sci..

[30] Mahabadi O., Cottrell B., Grasselli G. (2010). An example of realistic modelling of rock dynamics problems: Fem/dem simulation of dynamic brazilian test on barre granite. Rock Mech. Rock Eng..

[31] Mahabadi O., Grasselli G., Munjiza A. (2010). Y-GUI: A graphical user interface and pre-processor for the combined finite-discrete element code, Y2D, incorporating material heterogeneity. Comput. Geosci..

[32] Mahabadi O., Lisjak A., Munjiza A., Grasselli G. (2012). Y-geo: New combined finite-discrete element numerical code for geomechanical applications. Int. J. Geomech..

[33] Zhao Q., Lisjak A., Mahabadi O., Liu Q., Grasselli G. (2014). Numerical simulation of hydraulic fracturing and associated microseismicity using finite-discrete element method. J. Rock Mech. Geotech. Eng..

